# Monogamy relation of multi-qubit systems for squared Tsallis-*q* entanglement

**DOI:** 10.1038/srep28719

**Published:** 2016-06-27

**Authors:** Guang-Ming Yuan, Wei Song, Ming Yang, Da-Chuang Li, Jun-Long Zhao, Zhuo-Liang Cao

**Affiliations:** 1 School of Physics and Material Science, Anhui University, Hefei, 230601, China; 2 Institute for Quantum Control and Quantum Information; School of Electronic and Information Engineering, Hefei Normal University, Hefei 230601, China

## Abstract

Tsallis-*q* entanglement is a bipartite entanglement measure which is the generalization of entanglement of formation for *q* tending to 1. We first expand the range of *q* for the analytic formula of Tsallis-*q* entanglement. For 
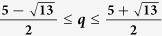
, we prove the monogamy relation in terms of the squared Tsallis-*q* entanglement for an arbitrary multi-qubit systems. It is shown that the multipartite entanglement indicator based on squared Tsallis-*q* entanglement still works well even when the indicator based on the squared concurrence loses its efficacy. We also show that the *μ*-th power of Tsallis-*q* entanglement satisfies the monogamy or polygamy inequalities for any three-qubit state.

Quantum entanglement as a physics resource for quantum communication and quantum information processing has been the subject of many recent studies in recent years^1^,^2^,^3^,^4^,^5^,^6^,^7^. The study of quantum entanglement from various view points has been a very active area and has led to many interesting results. Monogamy of entanglement(MOE)^8^ is an interesting property discovered recently in the context of multi-qubit entanglement, which means that quantum entanglement cannot be shared freely in multi-qubit quantum systems. The bipartite monogamy inequality was first proposed and proved by Coffman, Kundu and Wootters(CKW) in a three-qubit system^9^, and it is also named as CKW inequality:


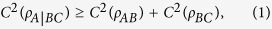


where 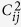 is the squared of concurrence between the pair *i* and *j*^10^. Later, the monogamy inequality was generalized into various entanglement measures such as continuous-variable entanglement^11^,^12^,^13^, squashed entanglement^14^,^15^,^16^, entanglement negativity^17^,^18^,^19^,^20^,^21^, Tsallis-*q* entanglement^22^,^23^, and Rényi-*α* entanglement^24^,^25^,^26^. The applications of monogamy relation include many fields of physics such as characterizing the entanglement structure in multipartite quantum systems^27^,^28^,^29^,^30^,^31^,^32^,^33^,^34^,^35^,^36^,^37^,^38^,^39^,^40^,^41^, the security proof in quantum cryptography^42^, the frustration effects observed in condensed matter physics^43^, and even black hole physics^43^,^44^,^45^,^46^,^47^,^48^. Originally, MOE was established in terms of the squared concurrence(SC). Analogously, Bai *et al*.^49^,^50^ have proved that the squared entanglement of formation(SEF) obeys the monogamy relation in arbitrary *N*-qubit mixed state. It should be noted that the entanglement of formation(EOF) itself does not satisfy the monogamy relation even for three-qubit pure states. The new monogamy relation in terms of SEF overcomes some flaws of the SC and can be used to detect all genuine multipartite entanglement for *N*-qubit systems.

On the other hand, Tsallis-*q* entanglement is also a well-defined entanglement measure which is the generalization of EOF. For *q* tending to 1, the Tsallis-*q* entanglement converges to the EOF. A natural question is whether the monogamy relation can be generalized to Tsallis-*q* entanglement. In fact, Kim has derived a monogamy relation in terms of Tsallis-*q* entanglement^22^. However, the result in ref. 22 fails in including EOF as a special case and only holds for 2 ≤ *q* ≤ 3. In this paper we further consider the monogamy relation in terms of the squared Tsallis-*q* entanglement(ST*q*E). Firstly we expand the range of *q* for the analytic formula of Tsallis-*q* entanglement. Then we prove a monogamy inequality of multi-qubit systems in terms of ST*q*E in an arbitrary *N*-qubit mixed state for 
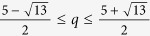
, which covers the case of EOF as a special case. Finally, we show that the *μ*-th power of the Tsallis-*q* entanglement satisfies the monogamy inequalities for three-qubit state.

## Results

### Analytic formula of Tsallis-*q* entanglement

Firstly we recall the definition of Tsallis-*q* entanglement introduced in ref. 22. For a bipartite pure state |*ψ*〉_*AB*_, the Tsallis-*q* entanglement is defined as


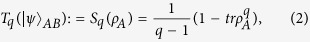


for any *q* > 0 and *q* ≠ 1, where *ρ*_*A*_ = *tr*_*B*_|*ψ*〉_*AB*_〈*ψ*| is the reduced density matrix by tracing over the subsystem *B*. For the case when *q* tends to 1, *T*_*q*_(*ρ*) converges to the von Neumann entropy, that is


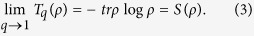


For a bipartite mixed state *ρ*_*AB*_, Tsallis-*q* entanglement is defined via the convex-roof extension


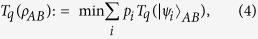


where the minimum is taken over all possible pure state decompositions of 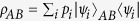.

In ref. 22, Kim has proved an analytic relationship between Tsallis-*q* entanglement and concurrence for 1 ≤ *q* ≤ 4 as follows


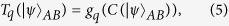


where the function *g*_*q*_(*x*) is defined as


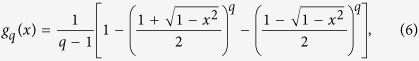


**Figure 1:**
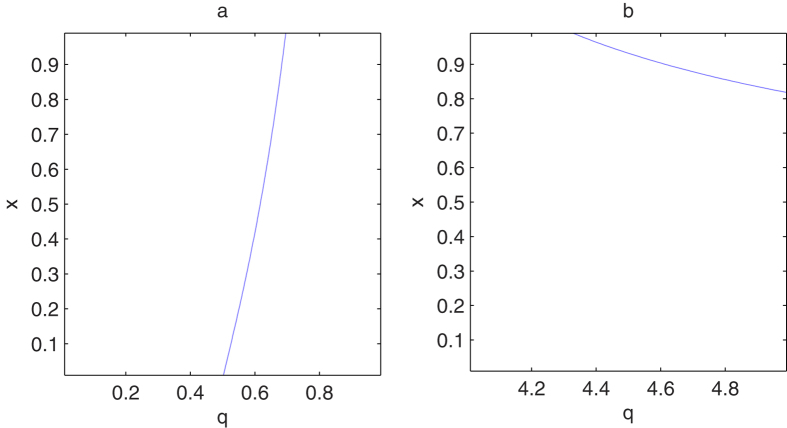
The plot of the dependence of *x* with *q* which satisfies the equation 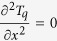 for (**a**) *q* ∈ (0, 1) and (**b**) *q* ∈ (4, 5) respectively.

According to the results in ref. 22, the analytic formula in Eq. (5) holds for any *q* such that *g*_*q*_(*x*) in Eq. (6) is monotonically increasing and convex. Next we shall generalize the range of *q* when the function *g*_*q*_(*x*) is convex and monotonically increasing with respect to *x*. The monotonicity and convexity of *g*_*q*_(*x*) follow from the nonnegativity of its first and second derivatives. After a direct calculation, we find that the first derivative of *g*_*q*_(*x*) with respect to *x* is always nonnegative for *q* ≥ 0^22^. Kim has also proved the nonnegative of the second-order derivative *g*_*q*_(*x*) for 1 ≤ *q* ≤ 4. We can further consider the second-order derivative of *g*_*q*_(*x*) beyond the region 1 ≤ *q* ≤ 4. We first analyze the nonnegative region for the second-order derivative *g*_*q*_(*x*) for *q* ∈ (0, 1). Numerical calculation shows that under the condition ∂^2^*T*_*q*_(*C*)/∂*x*^2^ = 0, the critical value of *x* increases monotonically with the parameter *q*. In Fig. 1(a), we plot the solution (*x*, *q*) to this critical condition, where for each fixed *x* there exists a value of *q* such that the second-order derivative of *T*_*q*_(*C*) is zero. Because *x* varying monotonically with *q*, we should only consider the condition ∂^2^*T*_*q*_(*C*)/∂*x*^2^ = 0 in the limit *x* → 1. When *x* = 1, we have


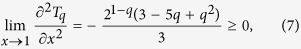


which gives the critical point 
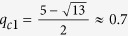
. When *q* > *q*_*c*1_, the second-order ∂^2^*T*_*q*_/∂*x*^2^ is always nonnegative. For *q* ∈ (4, 5), we find that the value of *x* decreases monotonically with respect to *q* as shown in Fig. 1(b). In order to determine the critical point we should only consider the condition ∂^2^*T*_*q*_/∂*x*^2^ = 0 in the limit *x* → 1. After direct calculation, we can obtain that the critical point 
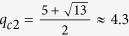
. When *q* < *q*_*c*2_, the second-order ∂^2^*T*_*q*_/∂*x*^2^ is always nonnegative. Combining with the previous results in ref. 22, we get that the second derivative of *g*_*q*_(*x*) is always a nonnegative function for 
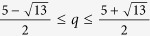
. Thus we have shown that the analytic formula of Tsallis-*q* entanglement in Eq. (5) holds for 
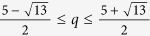
.

### Monogamy inequalities for ST*q*E in *N*-qubit systems

In the following we consider the monogamy properties of ST*q*E. Using the results presented in Methods, we can prove the main result of this paper.

For an arbitrary *N*-qubit mixed state 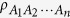, the squared Tsallis-*q* entanglement satisfies the monogamy relation


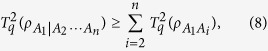


where 
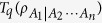
 quantifies the Tsallis-*q* entanglement in the partition *A*_1_|*A*_2_ ···*A*_*n*_ and 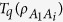 quantifies the one in two-qubit subsystem *A*_1_*A*_*i*_ with the parameter 
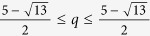
.

For proving the above inequality, we first analyze an *N*-qubit pure state 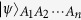. Under the partition *A*_1_|*A*_2_ ···*A*_*n*_, we have


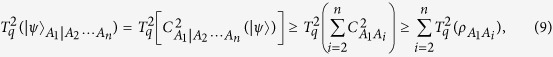


where in the first inequality we have used the monogamy relation of squared concurrence 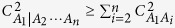 and the monotonically increasing property of 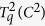 which has been proved in Methods, and the second inequality is due to the convex property of 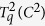 (The details for proving the convexity property can be seen from Methods).

Next, we prove the monogamy relation for an *N*-qubit mixed state 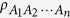. In this case, the formula of Tsallis-*q* entanglement cannot be applied to 
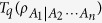
 since the subsystem *A*_2_ ···*A*_*n*_ is not a logic qubit in general. But we can still use the definition of Tsallis-*q* entanglement in Eq. (4). Thus, we have


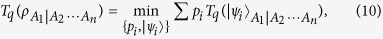


where the minimum is taken over all possible pure state decompositions {*p*_*i*_, |*ψ*_*i*_〉} of the mixed state 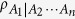. Under the optimal decomposition 
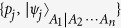
, we have


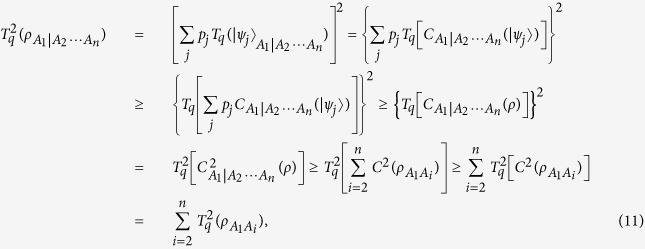


where in the second equality we have used the pure state formula of the Tsallis-*q* entanglement and taken the *T*_*q*_(*C*) as a function of the concurrence *C* for 
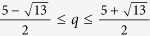
; the third inequality is due to that *T*_*q*_ is a monotonically increasing and convex function of the concurrence for 
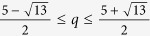
; the forth inequality is due to the convex property of concurrence for mixed state; and in the sixth and seventh inequalities we used the monotonically increasing and convex properties of 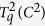 as a function of the squared concurrence for 
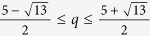
 (The details for illustrating the property of ST*q*E can be seen from Methods). Thus we have completed the proof of the monogamy inequalities for ST*q*E in *N*-qubit systems.

**Figure 2:**
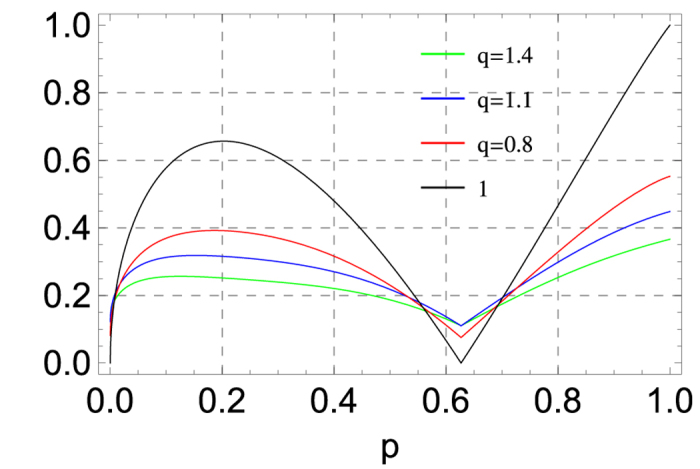
The indicator *τ*_*q*_ for the superposition state |*ψ*(*p*)〉 with *q* = 0.8 (red line), *q* = 1.1 (blue line), and *q* = 1.4 (green line). We also plot the three-tangle of |*ψ*(*p*)〉 with a black line.

As an application of the established monogamy relation in Eq. (8), we can construct the multipartite entanglement indicator 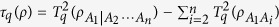 to detect the genuine multipartite entanglement. We consider a three-qubit pure state 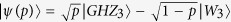, which is the superposition of a GHZ state and a W state with 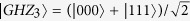 and 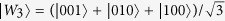. The three-tangle *τ* introduced in ref. 9 is defined as 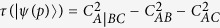. For the quantum state |*ψ*(*p*)〉, its three-tangle is 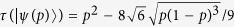 which has two zero points at *p*_1_ = 0 and *p*_2_ ≈ 0.627. On the other hand, we can directly calculate the value of *τ*_*q*_(|*ψ*(*p*)〉) since the Tsallis-*q* entanglement has an analytical formula for two-qubit quantum states. In Fig. 2 we plot the three-tangle and the indicator *τ*_*q*_ for the order *q* = 0.8, 1.1, 1.4. It is shown that the indicator *τ*_*q*_ is always positive for the different order *q* in contrast to the three-tangle *τ* having two zero points. Thus we have shown that the indicator in terms of Tsallis-*q* entanglement could detect the genuine entanglement in |*ψ*(*p*)〉 better than SC.

### Monogamy relation of the *μ*-th power of Tsallis-*q* entanglement

Finally, besides the squared Tsallis-*q* entanglement, we can further consider the monogamy relation of the *μ*-th power of Tsallis-*q* entanglement.

For any three-qubit state 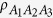, we can obtain


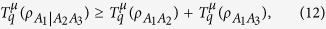


for all 
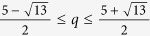
, *μ* ≥ 2.

For proving Eq. (12), we consider the three-qubit case, according to the monogamy relation (8), we have


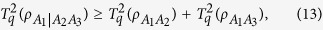


for any three-qubit state 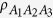 with 
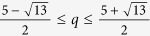
. Without loss of generality, assuming 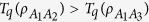, we can obtain


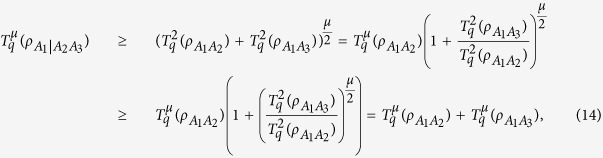


where the second inequality comes from the property (1 + *x*)^*t*^ ≥ 1 + *x*^*t*^ for *x* ≤ 1, *t* ≥ 1. If 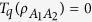 or 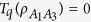, the inequality obviously holds.

Similarly, we have the following polygamy inequalities. For any three-qubit 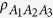, we have


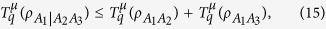


for all 
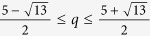
, *μ* ≤ 0.

For any three-qubit state 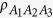 with 
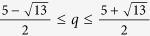
, we have


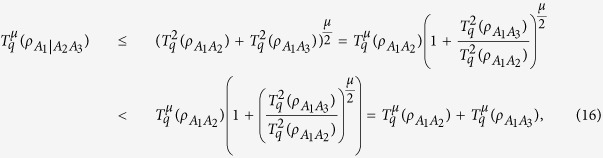


where in the second inequality we have used the inequality (1 + *x*)^*t*^ < 1 + *x*^*t*^ for *x* > 0, *t* ≤ 0.

## Discussion

In this paper we have generalized the analytic formula of Tsallis-*q* entanglement to the region 
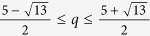
. Then we proved the monogamy relation in terms of ST*q*E for an arbitrary multi-qubit systems, which include previous result in terms of EOF as a special case. Based on the monogamy properties of Tsallis-*q* entanglement, we have shown that the corresponding indicator can work well even when the indicator based on the squared concurrence loses its efficacy. In addition, we considered the monogamy or polygamy relation of the *μ*-th power of Tsallis-*q* entanglement. One distinct advantage of our result is that infinitely many inequalities parameterized by *q* provides greater flexibility than previous monogamy relation in terms of EOF.

## Methods

### 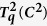 is a monotonically-increasing function of the squared concurrence *C* ^2^ for all *q* ≥ 0

Notice that Eq. (5) can also be written as


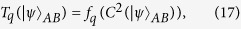


where the function *f*_*q*_(*x*) is defined as


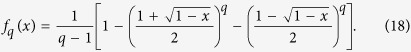


The squared Tsallis-*q* entanglement is a monotonically increasing function of *C*^2^ if the first-order derivative 
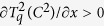
 with *x* = *C*^2^. By direct calculation, we have,


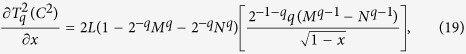


which is always nonnegative on 0 ≤ *x* ≤ 1 for all *q* ≥ 0, where *L* = 1/(*q* − 1)^2^, 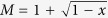, 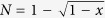, and the equality holds only at the boundary. Thus we get that 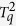 is a monotonically increasing function of *x* with *x* = *C*^2^.

### 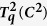 is a convex function of the squared concurrence *C* ^2^ for 
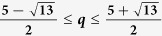


The convex property of the squared concurrence is satisfied if the second-order derivative 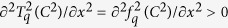 with *x* = *C*^2^. We first define a function 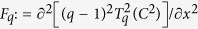 on the domain *D* = {(*x*, *q*)|0 ≤ *x* ≤ 1, 1 ≤ *q* ≤ 4}, then the nonnegativity of the second-order derivative 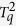 can be guaranteed by the nonnegativity of *F*_*q*_ since it varies with 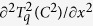 by a positive constant. After some deduction, we have


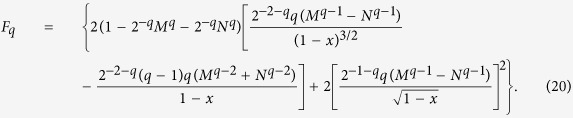


In order to prove the nonnegativity of *F*_*q*_, it is suffice to consider its maximum or minimum values on the domain *D*. The critical points of *F*_*q*_ satisfy the condition


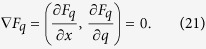


**Figure 3:**
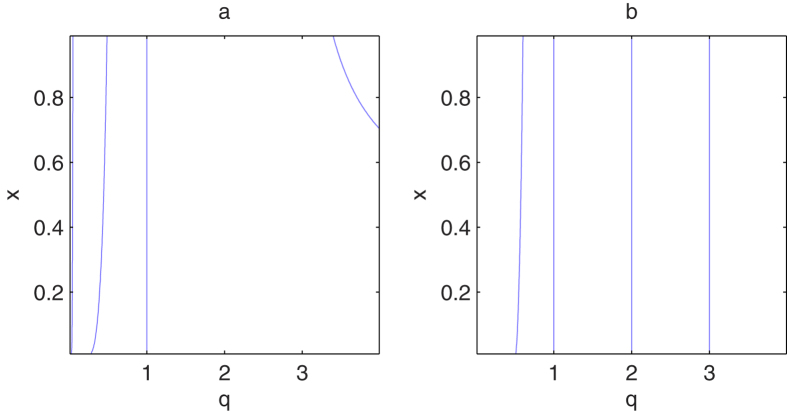
The plot of the dependence of *x* with *q* which satisfies the equation (**a**) 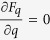 and (**b**) 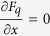 respectively.

In Fig. 3(a,b), we have plotted the value of *x* and *q* which satisfies the equation ∂*F*_*q*_/∂*q* = 0 and ∂*F*_*q*_/∂*x* = 0 respectively. Combining the results in Fig. 3(a,b), we find that the solution of the above equation is *q* = 1 which is one of the boundary of domain *D*. To ensure the nonnegative of *F*_*q*_, we should only consider the other two cases on the boundary of *F*_*q*_, i.e., *x* = 0 and *x* = 1.

For the case *x* = 0,


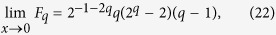


which is always nonnegative in the region *q* ∈ (1, 4).

**Figure 4:**
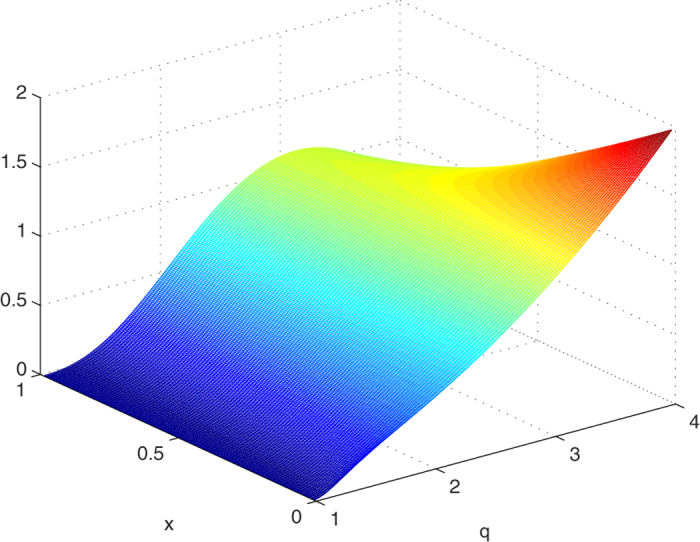
*F*_*q*_ is plotted as a function of *x* and *q* for 0 ≤ *x* ≤ 1, 1 ≤ *q* ≤ 4.

For the case when *x* = 1,


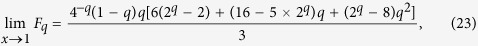


where Eq. (23) is always nonnegative for *q* = 1 and *q* = 4, and the first-order derivative of Eq. (23) increases first and then decreases for 1 ≤ *q* ≤ 4. Thus we prove that Eq. (23) is nonnegative in the region 1 ≤ *q* ≤ 4. Notice that *F*_*q*_ has no critical points in the interior of *D*, we conclude that *F*_*q*_ is always nonnegative for 1 ≤ *q* ≤ 4. The nonnegative of the *F*_*q*_ is also plotted in Fig. 4.

**Figure 5:**
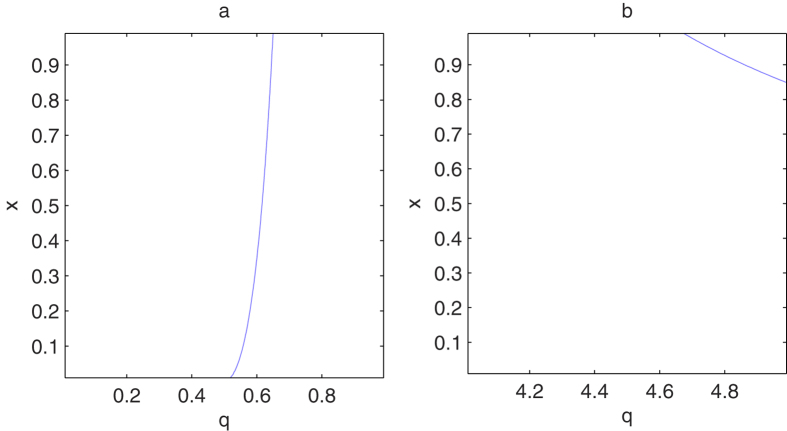
The plot of the dependence of *x* with *q* using the equation 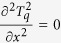 for (**a**) *q* ∈ (0, 1) and (**b**) *q* ∈ (4, 5) respectively.

Furthermore, we can consider the nonnegative region for the second-order derivative 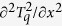 when *q* ranges in (0, 1). Under the condition 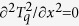, we find that the critical value of *x* increases monotonically with the parameter *q* ∈ (0, 1). In Fig. 5(a), we plot the solution (*x*, *q*) to the critical condition 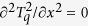 where for each fixed *x* there exists a value of *q* such that the second-order derivative of 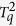 is zero. We should only consider the condition 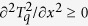 in the limit *x* → 1. In this case, we have


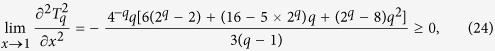


which gives the critical point *q*_*c*3_ ≈ 0.65. When *q* ≥ *q*_*c*3_, the second-order 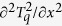 is always positive. Similarly, we can also analyze the nonnegative region for the second-order derivative 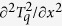 when *q* ranges in (4, 5). In Fig. 5(b), it is shown that the critical value of *x* decreases monotonically along with the parameter *q* ∈ (4, 5), and the critical point *q*_*c*4_ ≈ 4.65. When *q* ≤ *q*_*c*4_, the second-order 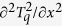 is always positive. Notice that the analytical formula of *T*_*q*_ is established only for 
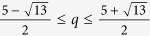
, we conclude that the second-order derivative 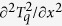 is positive for 
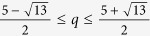
 which completes the proof of the convexity property of 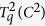 with the squared concurrence *C*^2^ for 
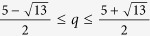
.

## Additional Information

**How to cite this article**: Yuan, G.-M. *et al*. Monogamy relation of multi-qubit systems for squared Tsallis-*q* entanglement. *Sci. Rep.* **6**, 28719; doi: 10.1038/srep28719 (2016).
